# ﻿Divergence of the freshwater sleeper, *Neodontobutishainanensis* (Chen, 1985) (Teleostei, Odontobutidae), in the Pearl River basin and on Hainan Island of southern China

**DOI:** 10.3897/zookeys.1197.110314

**Published:** 2024-04-17

**Authors:** Mingwei Zhou, Jianhong Xia, Chenhong Li

**Affiliations:** 1 Shanghai Universities Key Laboratory of Marine Animal Taxonomy and Evolution, Shanghai Ocean University, Shanghai 201306, China; 2 Engineering Research Center of Environmental DNA and Ecological Water Health Assessment, Shanghai Ocean University, Shanghai 201306, China; 3 Shanghai Natural History Museum, Branch of the Shanghai Science & Technology Museum, Shanghai 200041, China

**Keywords:** Gene flow, phylogeography, population structure, southern China

## Abstract

Study of divergence of freshwater fish populations between island and adjacent mainland areas can shed light on the phylogeographical relationships of these regions. *Neodontobutishainanensis* is a freshwater fish species restricted to Hainan Island and in Guangdong and Guangxi provinces in the southern mainland China. We examine the phylogenetic relationship and population structure of *N.hainanensis* based on 3,176 nuclear loci using a gene-capture method. STRUCTURE analysis and principal coordinate analyses (PCA) indicate that populations from Guangdong, Guangxi and Hainan are each distinct, except that some individuals of the Guangdong population share minor genetic components with individuals of the Guangxi population. In the concatenated gene tree, the Hainan population is grouped with the Guangdong population, but the coalescent tree groups the Hainan population as the sister to the Guangxi population. Finally, coalescent simulations confirmed the divergence pattern supported by the coalescent tree and revealed a one-way introgression from the Guangxi population to the Guangdong population, which can explain the discordant results supported by the concatenated and coalescent phylogenetic analyses. Due to recent decline of *N.hainanensis* populations and the genetic patterns in this species, as revealed in this study, the populations in the three areas should be treated as separate conservation units.

## ﻿Introduction

Hainan Island is the second largest island in China, with an area of 33,920 km^2^, and is in the northernmost part of the South China Sea (Zhu 2020). The northern corner of Hainan Island is separated from the Leizhou Peninsula of mainland China by the Qiongzhou Strait, whereas the western coast of Hainan Island is separated from Guangxi Zhuang Autonomous Region of the southern China and the northern Vietnam by the Beibu Gulf. Besides the Qiongzhou Strait and the Beibu Gulf, other geographic barriers, such as the Yunkai-Shiwan Mountains may also played a role in shaping population patterns of the region’s ichthyofauna. Hainan Island lies at 108°36'43"–111°2'31"E and 18°10'04"–20°9'40"N (Zhu 2020) and has a typical tropical climate. The island is home to about 138 native freshwater fish species belonging to 90 genera and 26 families (Xiong et al. 2018). There are 14 species endemic to Hainan Island, but most native species are also distributed on the adjacent mainland, such as in Guangdong and Guangxi (Xiong et al. 2018). The study of the population structure and phylogeography of these species would shed light on the biogeographical events on Hainan Island that have shaped the patterns of its freshwater ichthyofauna.

Although the Qiongzhou Straight is much narrower than the Beibu Gulf, both geographical and biogeographical evidence support the hypothesis that Hainan Island might have originated as part of the northeastern Indo-China Peninsula and adjacent Guangxi, China, but not from southeastern China, and particularly Guangdong (Zhu 2020). For example, flora of Hainan Island was found more closely related to that of Guangxi and northern Vietnam than to that of Guangdong; 110 genera of plants are shared with Guangxi and Vietnam, but only seven genera are exclusively shared with Guangdong (Zhu 2020). This pattern also is supported by population genetics of freshwater fishes, such as dwarf snakehead, *Channagachua* (Hamilton, 1822) (Wang et al. 2021) and white cloud mountain minnow, *Tanichthysalbonubes* Lin, 1932 (Zhao et al. 2018). Nonetheless, the population genetics of other freshwater fish species, which usually have a wide distribution, may support alternative scenarios (Tsao et al. 2016; Chen et al. 2017) and complex population history. For this reason, the choice of fish species is important in revealing different facets of biogeographical events that shaped the ichthyofauna.

The genus *Neodontobutis* (Gobiiformes, Odontobutidae) has three to six species (Iwata 2011; Zhou et al. 2022). *Neodontobutislani* Zhou, 2022 is reported only in Chongzuo City, southern Guangxi, China (Zhou et al. 2022). *Neodontobutishainanensis* is distributed in southern China, including restricted areas in Guangdong and Guangxi, and on Hainan Island (Wu and Zhong 2008). The other species are distributed in the northern Indo-China Peninsula (Iwata 2011; Zhou et al. 2022). *Neodontobutishainanensis* is a small, benthic species inhabiting hill streams and backwaters (Chen et al. 2002). Being a strict freshwater fish, of small size and with limited distribution and presumably low capacity for migration, *N.hainanensis* could be an appropriate species for testing phylogeographic relationships between Hainan Island and the adjacent mainland China.

We captured and sequenced 4,434 single-copy nuclear coding loci from the individuals of *N.hainanensis* collected from Hainan Island and Guangdong and Guangxi provinces, applyied a target-gene enrichment method, and performed phylogenetic reconstruction, STRUCTURE analysis, principal coordinate analysis (PCA) and coalescent simulations to infer the relationships of the populations from these three areas and possible historical events shaping the current genetic patterns in this species.

## ﻿Materials and methods

### ﻿Taxon sampling

**Figure 1.**
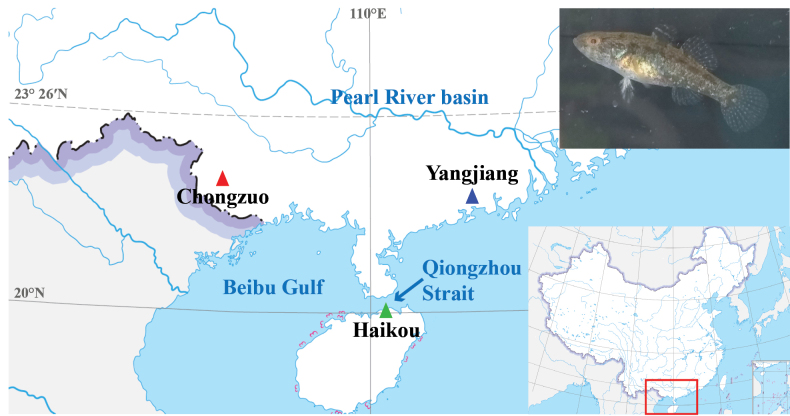
Sampling sites (triangles) for three populations of *Neodontobutishainanensis*: Yangjiang, Guangdong (blue), Chongzuo, Guangxi (red) and Haikou, Hainan (green).

Sixteen individuals of *N.hainanensis* were collected in total. Five specimens from Haikou, Hainan Island (20.0°N, 110.2°E), 5 specimens from Chongzuo, Zhuang Autonomous Region of Guangxi (22.8°N, 107.2°E), and six specimens from Yangjiang, Guangdong province (21.9°N, 112.1°E) (Table 1; Fig. 1). The specimens were deposited in the Fish Collection of Shanghai Ocean University (voucher numbers SOU1801005-1-5, SOU1801010-8-12, SOU1801011-2-3, SOU1801013, SOU1801014-1,-3,-4; contact person: Dr Ya Zhang, email: zhangya@shou.edu.cn). Fin clips or muscle tissue samples were preserved in ethanol for DNA extraction. Raw sequences of *Odontobutisyaluensis*, *Perccottusglenii*, and *Neodontobutislani* from previous studies (Li et al. 2018; Zhou et al. 2022) were used as outgroups (https://www.ncbi.nlm.nih.gov/, accessed February 2023, accession number SRP127338). Detailed sample information and sampling localities are shown in Table 1.

**Table 1.** Sampling information and localities of *Neodontobutishainanensis*.

Voucher number	Sample id	Location	Population id
SOU1801005-1	25461	Chongzuo, Guangxi	GX
SOU1801005-2	25462	Chongzuo, Guangxi	GX
SOU1801005-3	25463	Chongzuo, Guangxi	GX
SOU1801005-4	25464	Chongzuo, Guangxi	GX
SOU1801005-5	25465	Chongzuo, Guangxi	GX
SOU1801010-8	20278	Haikou, Hainan	HN
SOU1801010-9	20279	Haikou, Hainan	HN
SOU1801010-10	202710	Haikou, Hainan	HN
SOU1801010-11	202711	Haikou, Hainan	HN
SOU1801010-12	202712	Haikou, Hainan	HN
SOU1801011-2	CL1227_2	Yangjiang, Guangdong	GD
SOU1801011-3	CL1227_3	Yangjiang, Guangdong	GD
SOU1801013	CL1228	Yangjiang, Guangdong	GD
SOU1801014-1	CL1279_1	Yangjiang, Guangdong	GD
SOU1801014-3	CL1279_3	Yangjiang, Guangdong	GD
SOU1801014-4	CL1279_4	Yangjiang, Guangdong	GD
Outgroups	25913	Chongzuo, Guangxi	*Neodontobutislani*
	CL632_1	Harbin, Heilongjiang	*Perccottusglenii*
	CL1275_4	Dandong, Liaoning	*Odontobutisyaluensis*

### ﻿DNA extraction and target loci enrichment

Genomic DNA was extracted from tissue samples using an Ezup Column Animal Genomic DNA Purification Kit (Sangon, Shanghai, China). The concentration of extracted DNA was measured using a NanoDrop^TM^ 3300 fluorescence spectrophotometer, and the integrity of extracted DNA was visually checked using gel electrophoresis. A cross-species target loci enrichment method was used to enrich 4,434 coding regions of single-copy nuclear loci (Li et al. 2013). A set of gobioid-specific capture probes targeting the 4,434 loci were adopted from Li et al. (2018). Briefly, 300–1000 ng DNA were used for library preparation for gene enrichment according to Meyer and Kircher (2010), involving steps of shearing, blunt ending, ligation, and gap filling. Specific short sequences called “inline index” were added on 3’ end of both P5 and P7 adaptors to allow pooling different samples from the same population before the follow-up target enrichment steps and to track potential cross-contamination (Wang et al. 2022). After target enrichment, all samples were pooled in equimolar ratio for 2× 150 bp paired-end sequencing on an Illumina HiSeq X10 lane at Genewiz (Suzhou, Jiangsu, China).

### ﻿Data assemblage and multiple sequences alignment

Reads were assembled, aligned, and filtered following the ASSEXON pipeline, which includes a series of Perl scripts for processing target enrichment data (Yuan et al. 2019). Briefly, compressed raw reads were unzipped using gunzip_Files.pl. Reads from each sample were separated according to the combination of index sequences using demultiplex.pl. Adaptors and low-quality sequences were trimmed using trim_adaptor.pl, which invokes trim_galore v. 0.4.1 (http://www.bioinformatics.babraham.ac.uk/projects/trim_galore/). A set of 4,434 nuclear coding sequences of *Oreochromisniloticus* was used as a reference (Yuan et al. 2019). Reads were parsed to each locus according to the reference sequence and then assembled by using assemble.pl. Assembled sequences were uploaded to Mendeley Data (https://data.mendeley.com/datasets/8rgcmdxrmk/1). Output files include assembled coding regions of the loci were aligned by using mafft_aln.pl, which invokes Mafft v. 7.294b (Katoh and Standley 2013) for aligning. Finally, poorly aligned coding sequences were excluded by using filter.pl. Target enrichment results were summarized using statistics.pl (Yuan et al. 2019).

### ﻿Phylogenetic analysis

Aligned sequences were concatenated using a Perl script concat_loci.pl in the ASSEXON package (Yuan et al. 2019), then a Perl script, extract_DNAblocks.pl, was used to generate a partition scheme file by codon position. A concatenated maximum-likelihood tree including *N.hainanensis* from the three populations and the outgroups was constructed using IQ-TREE v. 1.7 (Minh et al. 2020) with 1,000 bootstrap replicates. The -spp option in IQ-TREE was applied to select the best model for each part according to the partition scheme file.

To infer the species tree, maximum-likelihood gene trees of all loci were reconstructed by using the Perl script construct_tree.pl in the ASSEXON package, which generated gene trees for each loci using RAxML v. 8.0.0 (Stamatakis 2014) under the GTRGAMMA model. Then, ASTRAL v. 4.10.6 (Mirarab et al. 2014) was used to generate a coalescent species tree from all gene trees. The resulting trees were visualized in FigTree v. 1.4.0 (Rambaut 2013).

### ﻿SNPs calling

Consensus sequences were generated from the aligned sequences of *N.hainanensis* using a Perl script consensus.pl in the ASSEXON package (Yuan et al. 2019). BWA v. 0.7.16 (Li and Durbin 2009) was used to build the index from the consensus sequence and align the trimmed raw reads of individual samples of *N.hainanensis* to the consensus sequence. GATK4 (McKenna et al. 2010) was used for SNP calling and filtering. VCFTOOLS v. 0.1.16 (Danecek et al. 2011) was used to exclude loci out of Hardy-Weinberg Equilibrium with a *p*-value < 0.001. A custom Perl script (vcftosnps.pl) (Cheng et al. 2019) was used to convert the VCF file output from GATK4 into NEXUS file and STRUCTURE file for PCA and STRUCTURE analysis. To avoid linkage disequilibrium, one SNP was randomly selected for each gene locus.

### ﻿Population structure analysis

Principal coordinate analyses (PCA) were conducted on the NEXUS file contained SNP data using TBTOOLS v. 2.03 (Chen et al. 2020). Values of PC1 and PC2 were plotted to show genetic clustering of individuals from different populations. Population structure was reconstructed using STRUCTURE v. 2.3.4 (Pritchard et al. 2000) with the STRUCTURE file contained SNP data. Length of burn-in period was set as 500. The number of MCMC reps after burn-in was set as 500,000. Candidate genetic cluster numbers (K value) was set from 1 to 3. Each run was repeated 40 times. The output result was compressed and uploaded to “STRUCTURE HARVESTER” (Earl and von Holdt 2012) to compute the best K value for plotting.

### ﻿Testing hypotheses of divergence and migration events

**Figure 2.**
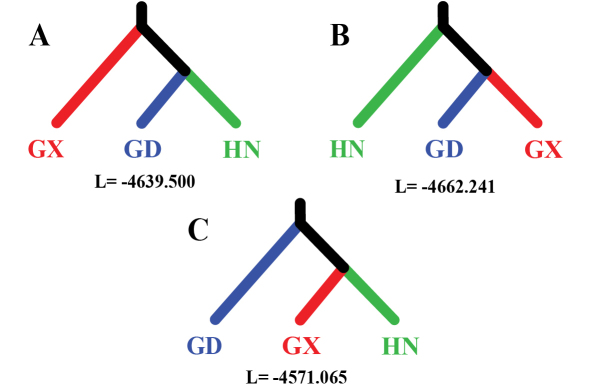
Three possible divergence models of *Neodontobutishainanensis*. GD: Guangdong population; GX: Guangxi population; HN: Hainan Island population. L represents Log likelihood values estimated using FASTSIMCOAL 2.7 for the three non-migration models.

FASTSIMCOAL v. 2.7 (Excoffier and Foll 2011) was used to test three possible divergence models of *N.hainanensis* as well as their divergence time (generations) and potential migration events (Fig. 2). FASTSIMCOAL v. 2.7 is versatile software that can estimate complex historical population events such as population resize, growth rates, and migration from site frequency spectrum (SFS). The VCF file (*.vcf) contains SNP data was converted to Arlequin file (*.arp) using PGDSPIDER v. 2.1.1.5 (Lischer and Excoffier 2012). ARLEQUIN v. 3.5 (Excoffier et al. 2007) was used to generate folded joint SFS (*.obs) from the Arlequin file. The model with the best likelihood was regarded as the optimal one to simulate the real divergent event. According to the PCA and STRUCTURE result, single-direction migration from Guangxi population to Guangdong population is proposed. Therefore, Migration matrix was added to the optimal model to estimate values of migration from Guangxi to Guangdong and the opposite direction respectively. All template files (*.tpl, see Suppl. material 1) that contain population parameters and estimation files (*.est, see Suppl. material 2) that contain unknown parameters for estimation were provided in Suppl. materials 1, 2.

## ﻿Results

### ﻿Read assembly and SNP calling

For each sample, 1,942–2,720 loci from the 4,434 targeted ones were obtained after assembling, aligning, and removing badly aligned sequences. A total of 3,176 loci were used for phylogenetic analysis and in the making of the consensus sequence. The length of the concatenated alignments was 583,539 bp with 29.13% gaps. A total of 3,493 SNP sites were detected through GATK calling and 996 sites were chosen subsequently for PCA, STRUCTURE analysis, and converted to SFS for FASTSIMCOAL 2 simulations.

### ﻿Phylogenetic analysis

**Figure 3.**
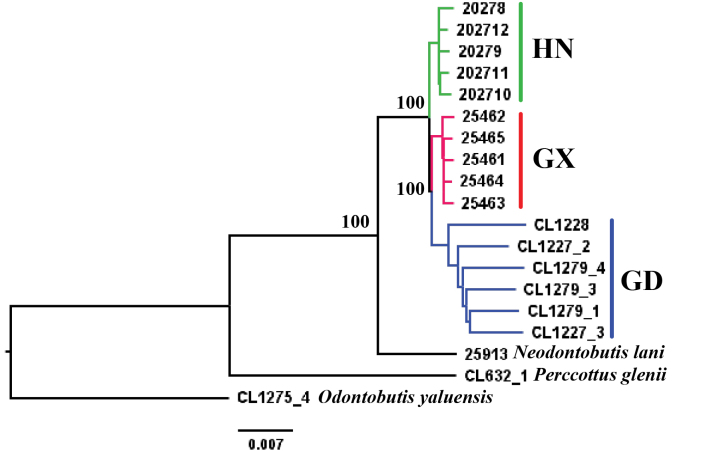
The concatenated maximum likelihood tree of *Neodontobutishainanensis*. GD: Guangdong population; GX: Guangxi population; HN: Hainan Island population.

**Figure 4.**
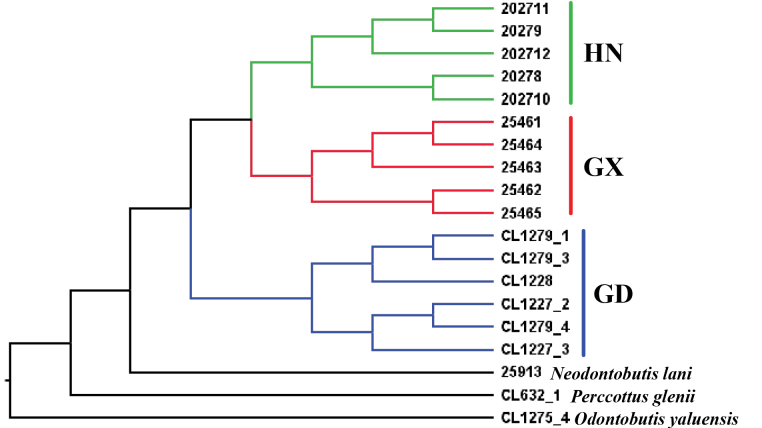
The ASTRAL coalescent tree of *Neodontobutishainanensis*. GD: Guangdong population; GX: Guangxi population; HN: Hainan Island population.

The concatenated maximum-likelihood tree is shown in Fig. 3. Individuals of the three populations form reciprocal monophyletic clades. The Guangdong population is sister to the Guangxi population, and then it is grouped with the population of Hainan Island. The ASTRAL coalescent species tree is shown in Fig. 4. In the coalescent tree, all three populations are monophyletic as well, but the Hainan Island population forms a clade with the Guangxi population, which is then sister to the Guangdong population.

### ﻿Population structure

**Figure 5.**
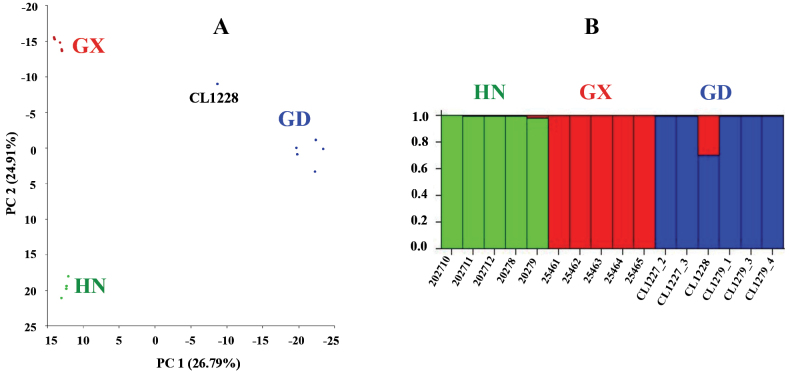
Result of principal component analysis (**A**) and population structure (**B**) on *Neodontobutishainanensis*. GD: Guangdong population; GX: Guangxi population; HN: Hainan Island population.

The PCA result was shown in Fig. 5A. All individuals from each population form a distinct cluster except for CL1228, which lies between the Guangdong population and Guangxi population. The STRUCTURE result is shown in Fig. 5B. Populations from Guangdong, Guangxi, and Hainan each formed distinct groups. However, some individuals in the Guangdong population, particularly CL1228 share some genetic components with individuals of Guangxi.

### ﻿Estimation on population historical events

**Figure 6.**
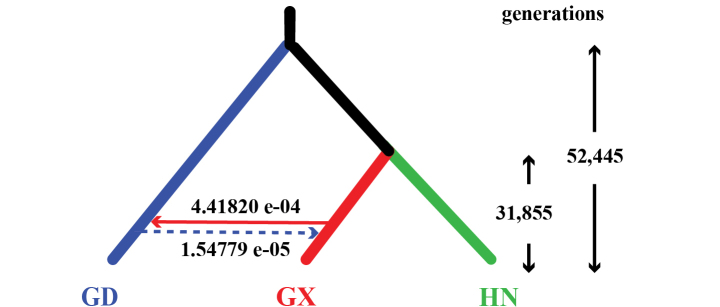
Historical population models simulate using FASTSIMCOAL 2.7. GD: Guangdong population; GX: Guangxi population; HN: Hainan Island population.

Log-likelihood values estimated by using FASTSIMCOAL v. 2.7 for the three non-migration models are shown in Fig. 2. The model which grouped the Guangxi population and the Hainan Island population as sister groups (Fig. 2C) showed best likelihood, indicating that it was the optimal model to explain real divergent events. According to the result of the STRUCTURE analysis, introgression from the Guangxi population to the Guangdong population is obvious, so the relevant migration option is added to the best non-migration model. The final historical population events estimated by FASTSIMCOAL v. 2.7 is shown in Fig. 6. The Guangdong population diverged from the common ancestor of *Neodontobutis* 52,445 generations ago, then the Hainan Island population and the Guangxi population diverged around 31,855 generations ago. The migration value from Guangxi populations to Guangdong population and the opposite direction were 4.41820 e^-4^ and 1.54779 e^-5^, respectively, indicating one-way introgression of *N.hainanensis* from the Guangxi population to the Guangdong population.

## ﻿Discussion

### ﻿High population differentiation of *Neodontobutishainanensis*

Both concatenated tree and species tree show that the three *N.hainanensis* populations are monophyletic. The PCA and STRUCTURE results show that despite some mixture in the Guangdong population, genetic compositions of the three populations are largely distinct. All results indicate that the three populations of *N.hainanensis* are highly differentiated. Because *N.hainanensis* is strictly freshwater fish of small body size, with a benthic habit, and presumably lacks planktonic eggs or a larval stage (Iwata et al. 2001; Iwata 2011), its capacity for migration may be limited. Similar phenomenon was also found in *Perccottusglenii* Dybowski, 1877 in northeastern China (Zhang et al. 2021), *Channagachua* around Beibu Gulf (Wang et al. 2021), and *Tanichthysalbonubes* in southern China and northern Vietnam (Zhao et al. 2018). In contrast, populations of *Hemiculterleucisculus* (Basilewsky, 1855) in Guangdong, in Guangxi, and on Hainan Island (Chen et al. 2017) do not show evident differentiation. The different patterns might be due to that *H.leucisculus* is an active pelagic fish with wider distribution, indicating its relatively high migration ability or incomplete lineage sorting due to large effective population size.

### ﻿Phylogenetic relationship of three populations of *Neodontobutishainanensis*

Although the three *N.hainanensis* populations were found to be monophyletic in both the concatenated tree and the coalescent tree, their phylogenetic relationship shows discordance. In the concatenated tree, the Guangxi population is sister of Guangdong population, but in the coalescent tree the Guangxi population is the sister to Hainan Island population. The results of FASTSIMCOAL v. 2.7 analysis corroborates the divergent history shown by the coalescent tree. Both PCA and STRUCTURE analyses indicate that migration occurred from the Guangxi population to the Guangdong population, which was confirmed by the FASTSIMCOAL v. 2.7 analyses. The migration from the Guangxi population to the Guangdong population might explain the discrepancy between the concatenated tree and the coalescent tree.

### ﻿Reconstruction of the divergent events in *Neodontobutishainanensis*

According to the results of species tree, PCA, STRUCTURE analysis, and FASTSIMCOAL v. 2.7 simulation, Indo-China Peninsula and the adjacent Guangxi are supposed to be at the center of diversity of the genus *Neodontobutis*, with two species (*N.hainanensis*, *N.lani*) distributed in Guangxi and presumably four species, *N.auarmus* (Vidthayanon, 1995), *N.tonkinensis* (Mai, 1978), *N.ngheanensis* Nguyen & Nguyen, 2011, and *N.macropectoralis* (Mai, 1978), found on the Indo-China Peninsula (Vietnam, Laos, and Thailand) (Iwata 2011; Zhou et al. 2022). We postulate that *N.hainanensis* might have originated in Guangxi and probably adjacent Hainan Island, which was connected with Guangxi and northern Vietnam during last glacial period due to lower sea levels (Yao et al. 2009). From there, *N.hainanensis* dispersed downstream of the Pearl River in Guangdong. Due to low migration ability of *N.hainanensis* and presumed vicariance events, the Guangdong population diverged from the common ancestor of Guangxi and Hainan population. After the sea level rose, Beibu Gulf formed, which resulted in divergence between the Guangxi population and the population of Hainan Island. A similar pattern of divergence is also observed in *Channagachua*, *Tanichthysalbonubes*, and *Opsariichthyshainanensis* Nichols & Pope, 1927, in which Hainan populations have closer relationships with Guangxi or Vietnamese populations than Guangdong populations (Zhao et al. 2018; Zhang et al. 2020; Wang et al. 2021). Nonetheless, populations of *Aphyocyprisnormalis* Nichols & Pope, 1927 and *Garraorientalis* Nichols, 1925 from northern Hainan Island are genetically closer to their Guangdong population (Chen and Jang-Liaw 2023; Yang et al. 2016), but the populations of southern or southwestern Hainan Island of these species were genetically distinct, indicating potentially independent origins, probably from the northern Indo-China Peninsula.

Besides the Qiongzhou Strait and the Beibu Gulf, the Yunkai-Shiwan Mountains may also be a significant barrier that shaped the genetic patterns of *N.hainanensis*. One-way introgression from the Guangxi population to the Guangdong population was detected from both STRUCTURE and FASTSIMCOAL v. 2.7 analyses. That may have caused by sporadic dispersal events, because the two populations are not in the same river system. Potential river-capture events await further study using species with similar distribution patterns.

Due to the recent population decline in *N.hainanensis*, we failed to collect more samples from each population. However, by utilizing genome-wide SNPs from thousands of loci in this study, we were able to mitigate the impact of having a limited number of individuals per population and still obtain valuable information. More samples from different populations of *N.hainanensis* as well as from other species of *Neodontobutis* from Vietnam would help to investigate the history of divergence in the genus. Excavating a complete fossil of the Odontobutidae also should help to precisely testing relevant geographical timeframe in southern China and on the Indo-China Peninsula. Because the recent decline of *N.hainanensis* and the distinct genetic patterns of the three populations revealed in this study, we recommend that the populations of *N.hainanensis* from Guangdong, Guangxi and Hainan should be treated as separate conservation units.

## ﻿Conclusion

*Neodontobutishainanensis* from Hainan Island, upstream and downstream of the Pearl River basin are distinct. The population of upstream Pearl River is sister group to the population of Hainan Island. One-way introgression from the population of upstream Pearl River to the population of downstream was supported by both population structure analysis and coalescent simulation.

## ﻿Additional information

### Author ORCIDs

Mingwei Zhou https://orcid.org/0000-0002-1182-8466

Jianhong Xia https://orcid.org/0009-0001-9615-4797

Chenhong Li https://orcid.org/0000-0003-3075-1756

### Data availability

All of the data that support the findings of this study are available in the main text or Supplementary Information.

## Supplementary materials

Supplementary material 1 A text file contains all template files (*.tpl) for FASTSIMCOAL2 simulations in this research Data type txt https://binary.pensoft.net/file/1027472This dataset is made available under the Open Database License (http://opendatacommons.org/licenses/odbl/1.0/). The Open Database License (ODbL) is a license agreement intended to allow users to freely share, modify, and use this Dataset while maintaining this same freedom for others, provided that the original source and author(s) are credited.Mingwei Zhou, Jianhong Xia, Chenhong Li

Supplementary material 2 A text file contains all estimation files (*.est) for FASTSIMCOAL2 simulations in this research Data type txt https://binary.pensoft.net/file/1027473This dataset is made available under the Open Database License (http://opendatacommons.org/licenses/odbl/1.0/). The Open Database License (ODbL) is a license agreement intended to allow users to freely share, modify, and use this Dataset while maintaining this same freedom for others, provided that the original source and author(s) are credited.Mingwei Zhou, Jianhong Xia, Chenhong Li
